# Optimizing the biodegradability and osteogenesis of biogenic collagen membrane via fluoride-modified polymer-induced liquid precursor process

**DOI:** 10.1080/14686996.2023.2186690

**Published:** 2023-03-13

**Authors:** Xiyan Li, Chuangji Li, Mengxi Su, Xinyi Zhong, Yihan Xing, Zhengjie Shan, Shoucheng Chen, Xingchen Liu, Xiayi Wu, Quan Liu, Ye Li, Shiyu Wu, Zhuofan Chen

**Affiliations:** a Hospital of Stomatology, Sun Yat-sen University, Guangzhou, China; b Guangdong Provincial Key Laboratory of Stomatology, Guangzhou, China; c Guanghua School of Stomatology, Sun Yat-sen University, Guangzhou, China

**Keywords:** Collagen membrane, degradation, amorphous calcium phosphate, polymer-induced liquid precursor, fluoride

## Abstract

Biogenic collagen membranes (BCM) have been widely used in guided bone regeneration (GBR) owing to their biodegradability during tissue integration. However, their relatively high degradation rate and lack of pro-osteogenic properties limit their clinical outcomes. It is of great importance to endow BCM with tailored degradation as well as pro-osteogenic properties. In this study, a fluoride-modified polymer-induced liquid precursor (PILP) based biomineralization strategy was used to convert the collagen membrane from an organic phase to an apatite-based inorganic phase, thus achieving enhanced anti-degradation performance as well as osteogenesis. As a result, three phases of collagen membranes were prepared. The original BCM in the organic phase induced the mildest inflammatory response and was mostly degraded after 4 weeks. The organic-inorganic mixture phase of the collagen membrane evoked a prominent inflammatory response owing to the fluoride-containing amorphous calcium phosphate (F-ACP) nanoparticles, resulting in active angiogenesis and fibrous encapsulation, whereas the inorganic phase induced a mild inflammatory response and degraded the least owing to the transition of F-ACP particles into calcium phosphate with high crystallinity. Effective control of ACP is key to building novel apatite-based barrier membranes. The current results may pave the way for the development of advanced apatite-based membranes with enhanced barrier performances.

## GRAPHICAL ABSTRACT

**Figure:**
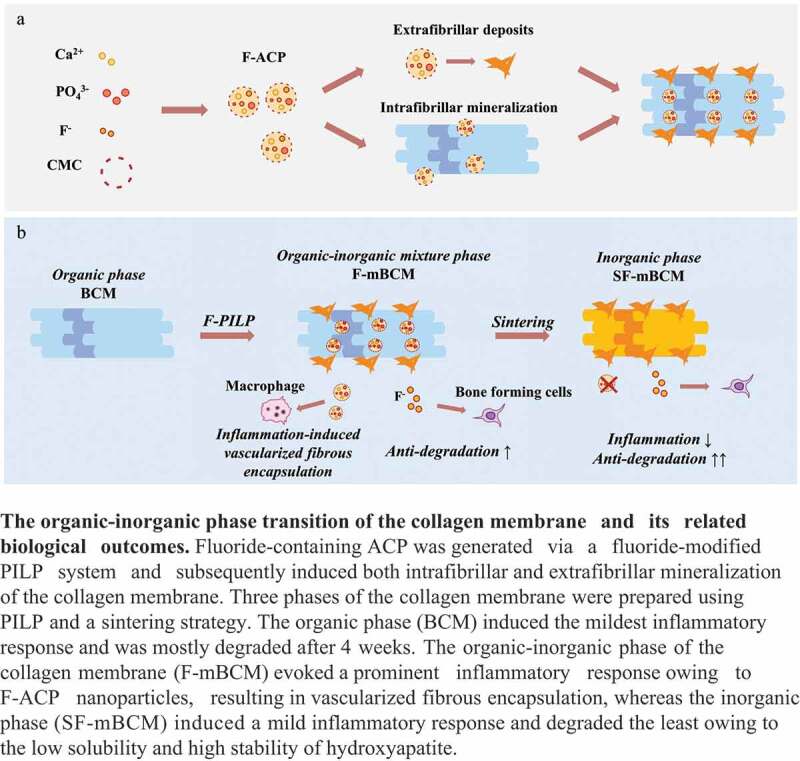

## 1. Introduction

Barrier membranes have been widely used in guided bone regeneration (GBR) and guided tissue regeneration (GTR). They create a stable healing space for bone defects by isolating soft tissue ingrowth [1]. Compared with other types of barrier membranes, biogenic collagen membranes (BCM) have a substantial advantage owing to their biodegradability during tissue integration. With a unique biologically derived smooth-rough membrane structure, the rough side of the BCM favors protein adsorption and cell attachment, whereas the smooth side prevents epithelial and fibroblast cells from entering the defected area [2]. However, this dual-functional effect gradually weakens with membrane degradation, a double-edged sword, which is a crucial property of BCM.

For clinical cases with large defects and requirements for long-term space maintenance, the degradation rate of the collagen membrane is relatively fast and may not provide sufficient barrier function. Following degradation, the space is filled with soft tissue owing to the lack of pro-osteogenic properties of the collagen membrane. These factors reduce the clinical effects of the GBR technique [3]. Therefore, it is very important to endow the collagen membrane with tailored degradation and pro-osteogenic properties.

At present, collagen membrane modification is mainly based on a cross-linking strategy combined with the addition of growth factors. The cross-linking technique using glutaraldehyde, 1-Ethyl-3-(3-dimethylaminopropyl) carbodiimide hydrochloride, and ribose has been shown to enhance the anti-degradation properties of collagen-based materials [4–6]. Growth factors have been used to endow the collagen membrane with additional biological functions, such as stromal cell-derived factor-1 alpha [7], basic fibroblast growth factor [8], interleukin-4, and interferon-gamma [9,10]. However, few cross-linking agents have low cytotoxicity [6], and the exogenous protein products result in issues, such as evoking additional oxidative stress on cells as well as the high cost and complex fabrication of protein additives [11]. Therefore, further improvements are required to optimize the properties of the collagen membrane.

The essential components of GBR are apatite-based bone substitutes and barrier membranes. As insoluble compounds with a stable inorganic crystal phase, apatite-based bone substitutes have a strong resistance to degradation. Our laboratory data and previous histological observations confirmed that the bone substitute in maxillary sinus elevation surgery can be stable for more than 14 years [12]. Accordingly, we propose a new strategy to convert the collagen membrane from an organic phase to an apatite-based inorganic phase, aiming to endow the membrane with enhanced anti-degradation performance via this phase transition. Moreover, nutrient elements can be introduced to the modified inorganic crystal phase of the membrane to promote bone regeneration while avoiding the application of cross-linking agents and exogenous proteins.

The polymer-induced liquid precursor (PILP) mineralization technique can translate a collagen template into an apatite-based inorganic phase in a bottom-up manner while retaining the microscopic morphology of the organic template [13,14]. Saxena et al. proposed a fluoride-modified PILP strategy in 2018, which combined the concepts of crystal phase transition and fluorine nutrient element modification [15]. In our previous work, biogenic hydroxyapatite was successfully modified by fluorine and improved osteogenesis and immune regulation performance, which was confirmed by both in vivo and in vitro results [16–19]. Thus, we believe that fluoride ions are a suitable nutrient element for imparting osteogenic properties to apatite-based materials and that this modified PILP strategy is a feasible solution to meet our demands.

As the feasibility and biological effect of this organic-inorganic phase transition of the collagen membrane remains unknown, we first developed a biogenic-derived collagen membrane (BCM) as the organic phase and a fluoride-containing biomineralized BCM (F-mBCM) as an organic-inorganic mixture phase. Their degradation and osteogenic performance and the underlying mechanism related to the immune response were then explored. On this basis, the membrane was further transformed to a fully inorganic phase (SF-mBCM) following a re-evaluation of its biological effects. This phase transition strategy of collagen membranes aims to combine the advantages of absorbable and non-absorbable barrier membranes. It proposed a new solution for optimizing the degradation property of the collagen membrane and may pave a new way for the development of advanced barrier membranes.

## 2. Materials and methods

### 2.1. Fabrication of BCM, F-mBCM, and SF-mBCM

#### 2.1.1. Preparation of BCM

Porcine peritonea were collected from the abdominal tissues of one-year-old black pigs following the removal of the fat tissue. Samples were divided into 4 cm × 4 cm pieces and decellularized using a chemical immersion procedure, as previously described [20,21]. In brief, the samples were treated with acid and alkali, hypertonic, and neutralization treatments, followed by dehydration and degreasing and were labeled as BCM. BCMs were fabricated after lyophilization in a vacuum freeze dryer (Christ, ALPHA 2-4, Germany) for 13 h.

#### 2.1.2. Preparation of F-mBCM

The biomimetic mineralization protocol is based on the principles of PILP [22]. Mineral solution A was prepared by mixing carboxymethyl chitosan (CMC) (920 mg/L) and CaCl_2_ (1332 mg/L) in 1000 mL of deionized water with stirring (600 rpm) until the CMC powder was completely dissolved. K_2_HPO_4_ (1460 mg/L) was dissolved in 1 L of deionized water to obtain mineral solution B. Solution B was slowly added to an equal amount of solution A under stirring. A series of fluoride concentrations were prepared (1250, 2500, and 5000 ppm F^−^) with deionized water and NaF. One milliliter of each NaF solution was added to 25 mL mineral solution B before mixing with solution A. The final F^−^ concentrations of the mixed mineral solutions were 25, 50, and 100 ppm. Samples were then soaked in 50 mL of the final mineral solution for 5 days, followed by daily replacement of the mineral solution and lyophilization to obtain F25 (25 ppm), F50 (50 ppm), and F100 (100 ppm). F25, F50, and F100 were also termed as F-mBCM.

#### 2.1.3. Preparation of SF-mBCM

Samples from F25 were soaked in 0.25 mol/L NaF solution for 24 h and lyophilized. The samples were then sintered at 800°C for 2 h in an air atmosphere at a heating rate of 10°C min^−1^ in a muffle furnace (SGM6812BK, Sigma Furnace Industry, China) to get SF-25/SF-mBCM.

### 2.2. Physicochemical characterization and biocompatibility evaluation

General views of all the samples, including the rough and smooth sides, were captured using an SLR camera (D160, Nikon, Japan). All the samples from each group were sputter-coated with gold and palladium. Both sides of the membrane were examined by scanning electron microscopy (SEM, SU8220, HITACHI, Japan). Energy dispersive spectrometry (EDS) was used to evaluate the elemental distribution of the membrane surface. Crystalline phases were examined using an X-ray powder diffractometer (XRD; Empyrean, Netherlands) and matched with the Joint Committee on Powder Diffraction Standards (JCPDS) database. Chemical bonding was characterized by a Fourier transform infrared spectrometer (FTIR, Nicolet6700-Continuum, Thermo Fisher, U.S.A.) with a resolution of 650–4000 cm^−1^. The thermal stability and proportion of the inorganic phase were tested by thermogravimetric analysis (TG), derivative thermogravimetry (DTG), and differential scanning calorimetry (DSC) with a synchronous thermal analyzer (Nicolet 6700, Thermo Fisher, U.S.A.) in an air atmosphere at a heating rate of 10°C/min, with the test temperature ranging from 22 to 1100°C.

The biocompatibility of the membrane was evaluated using Cell Counting Kit-8 (CCK-8) (Dojindo, Kumamoto, Japan) assay in Raw 264.7 cells (Chinese Academy of Sciences, Shanghai, China). Each sample was soaked in 2 mL complete medium for 24 h. The supernatant was collected and used as the conditioned medium. Cells were plated at a density of 2000 per well in 96-well plates and incubated with conditioned medium. The medium was replaced with a 10% CCK-8 solution for 45 min on days 1, 3, and 5. A microplate reader (TECAN, Thermo Scientific) was used to measure the absorbance at 450 nm.

To demonstrate the biomimetic mineralization process, collagen membranes under PILP were collected after 12 h and 5 days. Samples were embedded in resin and cut into cross sections (EM UC7, Leica, Germany). The Cu mesh was examined by transmission electron microscopy (TEM, HT7800, HITACHI, Japan; Talos-F200S, FEI, U.S.A.) at an accelerating voltage of 80 kV. Selected area electron diffraction (SAED) was used to detect the early characteristics of the crystalline phase after 12 h. To identify the fluoride-amorphous calcium phosphate (F-ACP) nanoparticles in the conditioned medium, the membrane was soaked in 1 mL water for 24 h. The supernatant was dispersed on a Cu mesh. Transmission electron microscopy (TEM, Tecnai G2 F30, FEI, America) and SAED were used to examine F-ACP and its crystalline phase.

### 2.3. Animal study

#### 2.3.1. Animal surgery

All experiments were approved by the Institutional Animal Care and Use Committee, Sun Yat-sen University, and performed according to the animal use protocol (No.SYSU-IACUC-2022-000673).

For the rat calvarial defect model: Eight-week-old male Sprague-Dawley (SD) rats (220–250 g) were used in this study. General anesthesia was administered before surgery using Zoletil®50 containing zolazepam and tiletamine (VIRBAC, France) at a dose of 60 mg/kg. The sample was soaked in 1 mL of tail blood for 15 min. The rat parietal bone was carefully exposed and two 5-mm critical-sized calvarial defects were prepared using a sterile trephine bur. The calvarial defects were covered with a membrane. In the blank group, the defect was solely filled with a blood clot. The incision was carefully sutured without moving the membrane. Each group contained at least 3 biological replicates. The animals were sacrificed after four weeks. For the rat subcutaneous model, pieces of 1 × 1 cm membranes (F25 and SF25) were inserted into the subcutaneous pouch on the left and right of the dorsum under general anesthesia. The rats were euthanized 3 days after the implantation surgery for subsequent analysis. The parietal bone with the defect area and the subcutaneous pouch with membranes were removed from the animal and fixed with paraformaldehyde for 24 h. Micro-CT (Bruker SkyScan 1276, 85 kV, 200 μA, 15 μm resolution) was used to evaluate osteogenesis and the residual amount of the membrane for the calvarial defects.

#### 2.3.2. Histological section staining

After micro-CT evaluation, the samples were decalcified using 4% ethylenediaminetetraacetic acid for 4 weeks. The samples were then embedded in paraffin and cut into 4-µm slices using a rotary microtome (Leica, RM2255, Germany), followed by dewaxing and hydration. Hematoxylin and eosin (H&E) and Masson’s trichrome staining were used to evaluate the biocompatibility, integration, and degradation properties of the membrane. For H&E staining, the cell nuclei were stained with Mayer’s hematoxylin (Sigma-Aldrich, St. Louis, MO, U.S.A.), whereas the cell plasma and extracellular matrix were stained with eosin (Sigma-Aldrich). Masson’s trichrome staining (G1340, Solarbio, China) was performed according to the manufacturer’s instructions. Muscle fibers were stained red, and collagen fibers were stained green/blue. Immunohistochemistry (IHC) staining of α-smooth muscle actin (α-SMA) was applied. Endogenous peroxidase activity was eliminated by incubating in 3% H_2_O_2_ for 15 min. The slides were blocked for 1 h and incubated with α-SMA antibodies (1:1000; Abcam, Cambridge, MA, U.S.A.) overnight at 4°C. The sections were then incubated with a secondary antibody (Gene Tech, Shanghai, China) for 30 min at room temperature. The antibody complexes were visualized by diaminobenzidine (DAB) solution (Gene Tech, Shanghai, China) and counterstained with Mayer’s hematoxylin for 90 s. Tartrate-resistant acid phosphatase (TRAP) (Servicebio, Wuhan, China) staining was performed to identify TRAP+ MNGCs. The sections were rehydrated and incubated with TRAP staining solution for 1 h at 37°C followed by staining with hematoxylin. Images were captured using a microscope slide scanner (Aperio AT2; Leica, Germany). For semi-quantitative analysis, the number of multinucleated giant cells (MNGC) around the residual material and the number of vessels were counted from three non-overlapping views (1 mm^2^) using Aperio ImageScope 12.3 (Leica Biosystems, Germany). The percentage of residual membrane area was calculated in three non-overlapping views (1 mm^2^) (Supplementary Figure S1).

### 2.4. Cytology study in vitro

#### 2.4.1. Cell culture

Raw 264.7 cells were cultured under standard conditions (37°C, with 5% CO_2_ atmosphere and 100% relative humidity) in Dulbecco’s modified Eagle’s medium (Thermo Scientific, Australia) containing 5% fetal bovine serum (Thermo Scientific) and 1% (v/v) penicillin/streptomycin (Thermo Scientific). The culture medium was changed every two days. The cells were gently scraped off and passaged once they reached 80% confluency.

#### 2.4.2. RNA extraction and quantitative real-time PCR (RT-PCR)

Cells were stimulated with the conditioned medium of collagen membranes for 24 h. Total RNA was extracted using TRIzol reagent (Life Technologies, U.S.A.) following the manufacturer’s instructions. RNA was reverse-transcribed into single-stranded cDNA using the TaKaRa PrimeScript^TM^ Master Mix (Perfect Real Time Kit; Takara, Japan). RT-PCR was performed on an equivalent quantity of cDNA using Hieff® qPCR SYBR Green Master Mix (No Rox; Yeasen, Shanghai, China) according to the manufacturer’s instructions. The relative mRNA expression of inflammation-related genes [interleukin 1 beta (*IL-1β*), tumor necrosis factor-alpha (*TNF-α*), interleukin- 6 (*IL-6*), nuclear factor kappa-B1 (*NFκB1*), and interleukin 1 receptor antagonist (*IL-1rn*)], osteoclastogenesis-related genes [macrophage colony-stimulating factor (*MCSF*)], angiogenic factors [vascular endothelial growth factor (*VEGF*)], osteogenesis- and fibrosis-related genes [transforming growth factor-β1 (*TGF-β1*) and *TGF-β3*)], and collagenase [matrix metalloprotein 9, (*MMP9*)] were detected in the F-mBCM and blank groups. The relative mRNA expression of inflammation-related genes (*IL-1β*, *TNF-α*, *IL-6*, *NFκB1*, and *IL-1rn*), osteoclastogenesis-related genes (*MCSF*), osteogenesis- and fibrosis-related genes [*TGF-β1*, *TGF-β3*, and oncostatin M, (*OSM*)], and collagenase (*MMP9*) were detected in the SF25 and blank groups. The 2^−ΔΔCt^ method was used to calculate relative gene expression after normalization against the housekeeping gene glyceraldehyde-3-phosphate dehydrogenase (*GAPDH*). Primers used in this study are listed in Supplementary Table S1.

### 2.5. Statistical analysis

GraphPad Prism (version 8.0.2) software was used for statistical analysis. All data were shown as the mean ± standard deviation (SD). The one-factor analysis of variance (ANOVA) comparison test was used to evaluate the differences among groups followed by Tukey (multiple comparisons) post hoc test. For all tests, significance was considered at *P* < 0.05. The significant difference between groups was marked as follows: *, *P* < 0.05, **, *P* < 0.01, ***, *P* < 0.001, ****, *P* < 0.0001. Data without * were not significantly different.

## 3. Results

### 3.1. Physicochemical properties and biocompatibility of F-mBCM

**Figure 1.**
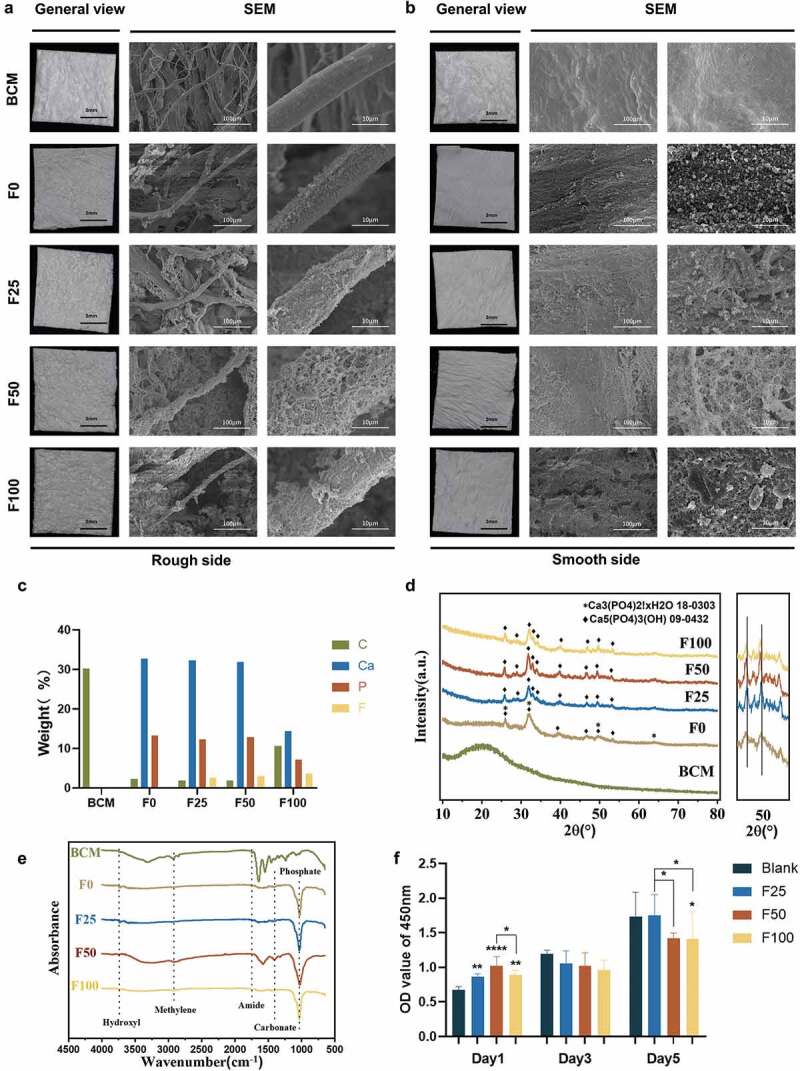
Physicochemical properties and biocompatibility of BCM, F0, F25, F50, and F100. (a) General view and SEM of BCM, F0, F25, F50, and F100 of the rough side. (b) General view and SEM of BCM, F0, F25, F50, and F100 of the smooth side. (c) EDS shows the elemental distribution of collagen fiber in (a). (d) XRD spectra of BCM, F0, F25, F50, and F100. (e) FTIR spectra of BCM, F0, F25, F50, and F100. (f) Cytotoxicity test of F-mBCM in Raw 264.7 by CCK-8 assay. * Significant differences compared with the blank group or between two groups. *, P < 0.05, **, P < 0.01, ***, P < 0.001, ****, P < 0.0001, data without * were not significantly different (NS). Error bars represented mean ± SD.

The membrane morphologies were evaluated using an SLR camera and SEM. The general view of biomineralized F-mBCM is consistent with that of BCM. SEM showed extrafibrillar deposits on the fiber surface in the F-mBCM groups, whereas the number of deposits increased with an increase in the fluoride ion content (Figure 1(a,b)). EDS detected calcium and phosphate elements in the F0–F100 samples, indicating mineralization in collagen membranes. Fluoride was detected in the F25–F100 samples, indicating the successful doping of fluoride onto the biomineralized collagen membranes (Figure 1(c)). The crystal phase of F-mBCM was composed of Ca_3_(PO4)_2_. xH_2_O, and Ca_5_(PO_4_)_3_(OH), according to the XRD results. In groups F25, F50, and F100, as the degree of fluorination increased, the shift in the peak value was more pronounced in the enlargement of 50° (2θ), which indicated the successful doping of fluorine (Figure 1(d)). The FTIR spectrum of the BCM group showed obvious absorption peaks of the collagen amide bands at 1037, 1240, and 1552 cm^−2^, respectively. After mineralization, the absorption peaks of the amide disappeared, and the absorption peaks of phosphate appeared at 1030 cm^−2^, indicating the existence of calcium phosphate. Amide absorption peaks of amide, methylene and hydroxyl were observed at 1750, 2930, and 3730 cm^−2^, respectively, among all mineralized groups (Figure 1(e)). A decrease in cell vitality was observed in F100 after 5 days of stimulation in the cytotoxicity test, whereas the other samples showed no toxic effects after 5 days of treatment (Figure 1(f)).

### 3.2. Formation of biomimetic mineralized collagen fibers

**Figure 2.**
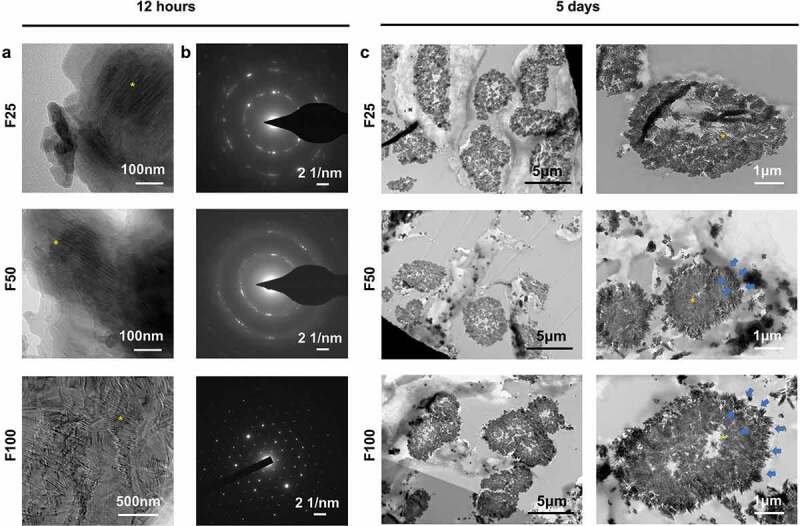
TEM showed the formation of biomimetic mineralized collagen fibers. (a) TEM showed the initial intrafibrillar mineralization process of collagen fibers after biomineralization for 12 h. (b) SAED patterns of (a). (c) TEM showed the cross-section of collagen fibers after biomineralization for 5 days.

TEM was used to detect the intrafibrillar mineralization structure of F-mBCM. After 12 h of mineralization, TEM captured partially mineralized collagen in F25 and F50 with hierarchical intrafibrillar banding, whereas heavily mineralized fibrils were found in F100 (Figure 2(a)). The SAED patterns showed a polycrystalline morphology in the F25 and F50 groups, whereas F100 showed a monocrystalline morphology pattern (Figure 2(b)). After 5 days of mineralization, the cross-section of the collagen fibers was observed using TEM. Both intrafibrillar mineralization (yellow asterisks) and extrafibrillar mineralization (blue arrows) were observed around the fibers. The amount of extrafibrillar deposits (blue arrows) around the collagen fibers on the fiber surface increased as the fluoride ion content increased (Figure 2(c)).

### 3.3. Osteogenesis, degradation, and integration evaluation of F-mBCM

**Figure 3.**
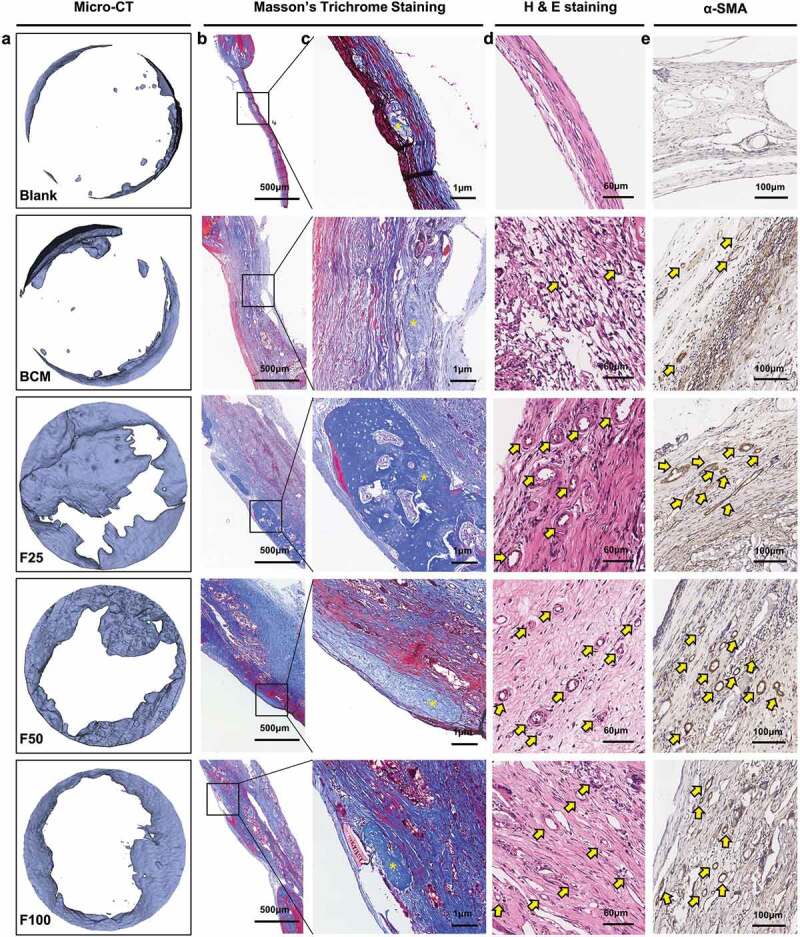
Effects of F-mBCM on osteogenesis at the calvarial defect at 4 weeks following surgery. (a) Micro-CT showed the new bone formation at the calvarial defect without barriers. (b, c) Masson’s trichrome showed the (b) overview of calvarial defect areas and (c) higher magnification of new bone tissue (yellow asterisks). (d) H&E staining and (e) α-SMA staining showed that few blood vessels were observed in blank and BCM groups, whereas more were found in F-mBCM groups (yellow arrows).

**Figure 4.**
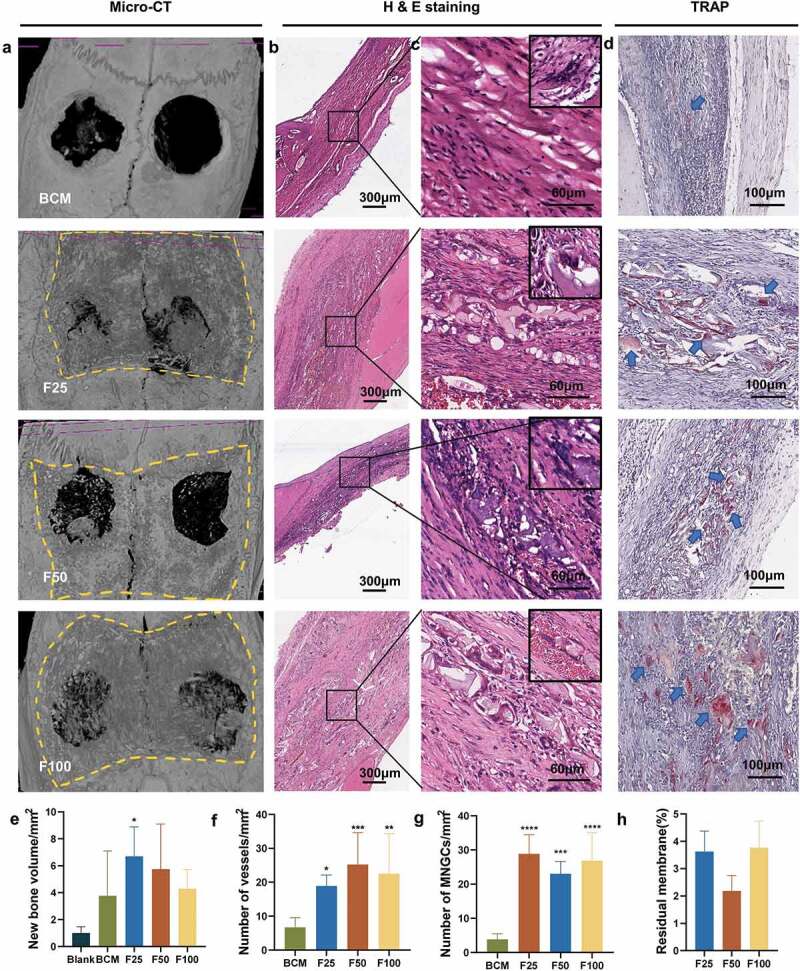
Degradation and integration properties of F-mBCM at the calvarial defect at 4 weeks following surgery. (a) Micro-CT revealed the residual membrane of F-mBCM over the calvarial defect (yellow area). (b, c) H&E staining showed that the residual membrane was surrounded by fibrous tissue and MNGCs. (d) TRAP staining showed the presence of TRAP+ MNGCs (blue arrows). Semi-quantitative analysis of (e) new bone volume, (f) the number of vessels, (g) the number of MNGCs, and (h) the percentage of residual membrane area. * Significant differences compared with the blank or BCM groups. *, P < 0.05, **, P < 0.01, ***, P < 0.001, ****, P < 0.0001, data without * were not significantly different (NS). Error bars represented mean ± SD.

Biological performance, including osteogenesis, degradation, and integration properties, was evaluated using a rat calvarial defect model. Micro-CT and Masson’s trichrome staining showed that the defects were partially repaired with new bone tissue in the BCM, F25, F50, and F100 groups, whereas few new bone islands were observed in the blank group (Figures 3(a,b) and 4(e)). Few blood vessels were observed in the blank and BCM groups, whereas more blood vessels with fibrous formation were found in all the F-mBCM groups around the membrane (Figures 3(d,e) and 4(f)). The contour of the residual mineralized membrane covered the bone defects, suggesting that the membrane position remained stable without deviation during the entire animal experiment (Figure 4(a)). In the BCM group, the membrane was mostly degraded and replaced with fibrous connective tissue. In the F-mBCM group, mineralized membrane fragments were retained with vascularized fibrous encapsulation formed around them (Figure 4(b)). TRAP+ MNGCs were observed around the residual fragments in the F-mBCM group, which were significantly higher than those in the BCM group (Figure 4(d,g)). No significant difference was found in the amount of residual membrane among F-mBCM groups (Figure 4(h), Supplementary Figure S1).

### 3.4. Effects of F-mBCM on modulating the immune response of macrophages

**Figure 5.**
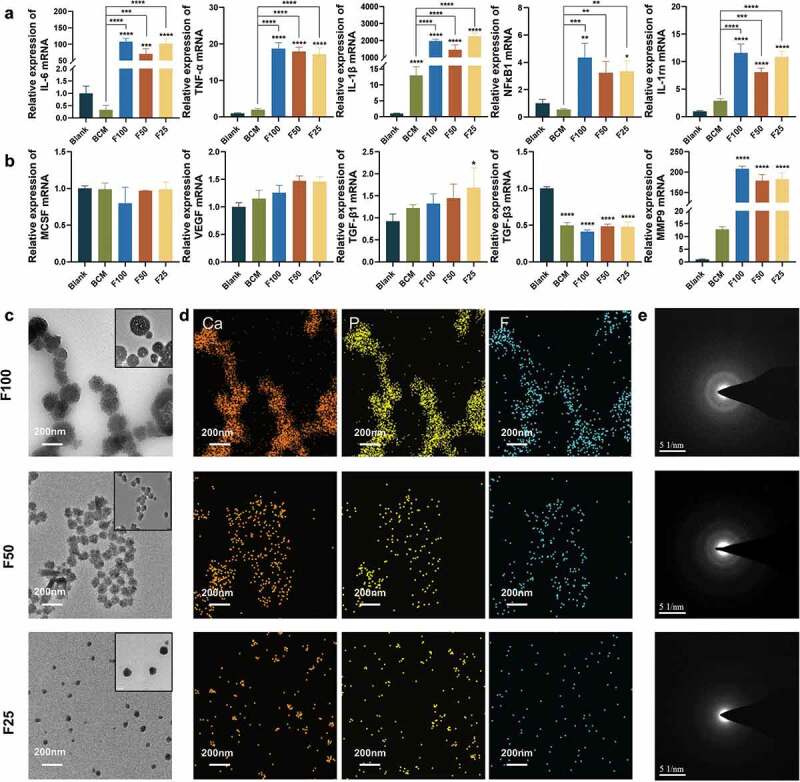
The effect of F-mBCM on modulating immune response of Raw 264.7 in vitro. Relative mRNA expression of (a) inflammation-related genes (*IL-1β*, *TNF-α*, *IL-6*, *NFκB1*, and *IL-1rn*). (b) Osteoclast genesis-related genes (*MCSF*), angiogenic factor (*VEGF*), osteogenesis- and fibrosis-related genes (*TGF-β1* and *TGF-β3*), and collagenase (*MMP9*). (c) TEM, (d EDS mapping, and (e) SAED showing the presence of F-ACP nanoparticles in the extracts of F-mBCM. * Significant differences compared with the blank group. *, P < 0.05, **, P < 0.01, ***, P < 0.001, ****, P < 0.0001, data without * were not significantly different (NS). Error bars represented mean ± SD.

Because significant degradation of F-mBCM was observed in vivo, the effects of F-mBCM on the immune response of macrophages were further examined. Inflammation-related genes (*IL-6*, *TNF-α*, *IL-1β*, *NFκB1*, and *IL-1rn*) were significantly upregulated in the BCM and blank groups (Figure 5(a)). The expression of osteoclastogenesis-related genes (*MCSF*) was not significantly different from that of the control group. The expression of the angiogenic factor (*VEGF*) was upregulated in the F-mBCM groups without a significant difference. The fibrosis-enhancing factor, *TGF-β1*, was significantly upregulated in the F25 group, whereas *TGF-β3* was significantly downregulated in both the BCM and F-mBCM groups. Collagenase (*MMP9*) was significantly upregulated in all F-mBCM groups (Figure 5(b)).

To explore the causes of barrier membrane inflammation, we conducted a detailed literature review and found that amorphous calcium phosphate (ACP) nanoparticles are an important potential inflammatory factor. We detected the presence of fluoride-containing ACP (F-ACP) nanoparticles in the material extracts using TEM. The particle size increased from 30 to 200 nm with an increase in the fluoride ion concentration in the PILP system (Figure 5(c)). The EDS analysis showed that the elemental composition of the particles was calcium, phosphate, and fluoride (Figure 5(d)). SAED analysis showed that the crystalline phase was amorphous in F25, F50, and F100 (Figure 5(e)).

### 3.5. Physicochemical properties and biocompatibility of SF-mBCM/SF25

**Figure 6.**
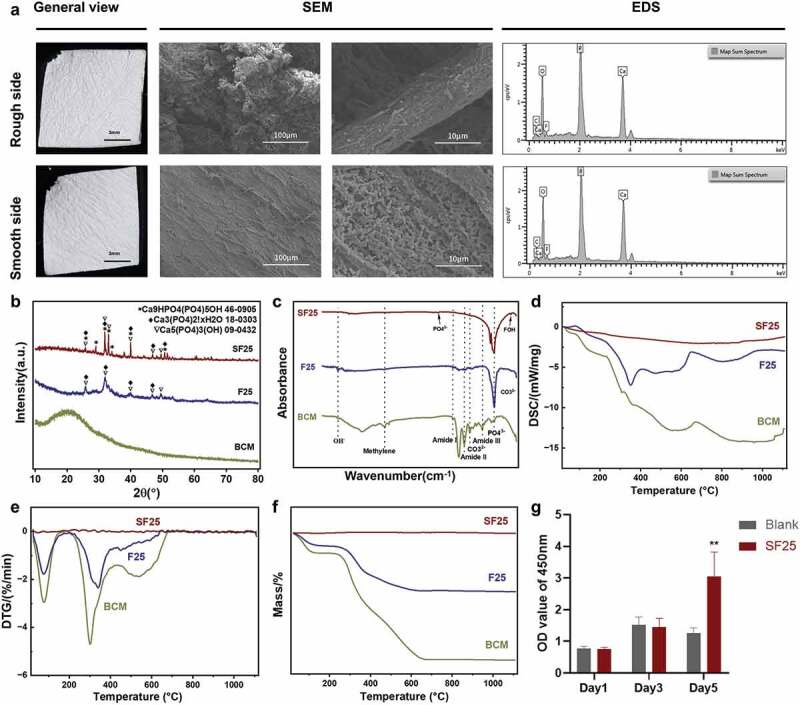
Physicochemical properties and biocompatibility of SF25. (a) General view, SEM, and element distribution of SF25. (b) XRD of BCM, F25, and SF25. (c) FTIR of BCM, F25, and SF25. (d) Cytotoxicity test of SF25 in Raw 264.7 by CCK-8 assay. (e) DTG patterns. (f) TG patterns. (g) DSC patterns.

High-temperature sintering was performed to stabilize the F-ACP nanoparticles on F-mBCM. After sintering at 800°C for 2 h, the general view of the generated SF25 was consistent with that of F25. SEM showed that the structure of the collagen membrane was well-preserved on both the rough and smooth sides, whereas the mineralized substances on the membrane surface partially fused to form rod-like shapes under high-temperature sintering. EDS analysis showed that the elemental composition of SF25 was calcium, phosphate, and fluoride (Figure 6(a)). XRD revealed a higher and narrower peak in the SF25 group than that in the F25 group, indicating the higher crystallinity of SF25 (Figure 6(b)). The FTIR spectrum of SF25 showed an absorption peak at 746 cm^−2^, which is possibly owing to the interaction between OH^−^ and F^−^ as further evidence of fluorine incorporation. The characteristic phosphate group bands at 1097 cm^−2^ and 1035 cm^−2^ were visible. This indicates a fluorohydroxyapatite composition in SF25 (Figure 6(c)). DSC results showed that a cold crystal peak was observed in the F25 group at 350°C, whereas an endothermic melting peak was observed in both the F25 and BCM groups. No obvious peaks were observed in the SF25 group (Figure 6(d)). The weight loss rates of SF25 differed from those of F25 and BCM. The latter two showed a peak at 75°C, representing the volatilization of water. Peaks of F25 and BCM at 340 and 300°C, respectively, represent the decomposition of organic components. No peak was found for SF25, indicating the absence of an organic component (Figure 6(e,f)). No toxic effects were found after 5 days of culture of Raw 264.7. SF25 significantly increased the cell viability on day 5 (Figure 6(g)).

### 3.6. Effects of SF25 on modulating the immune response

**Figure 7.**
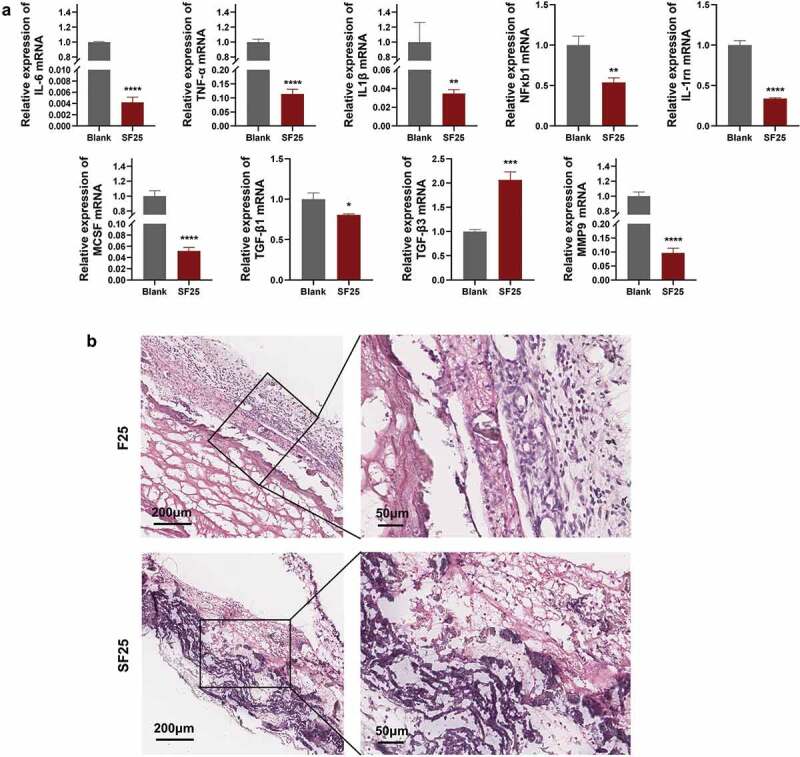
Effects of SF25 on modulating the immune response *in vitro* and *in vivo*. (a) Relative mRNA expression of macrophage inflammation-related genes (*IL-1β*, *TNF-α*, *IL-6*, *NF-κB1*, and *IL-1rn*), osteoclastogenesis-related gene (*MCSF*), fibrosis-related genes (*TGF-β1* and *TGF-β3*,) and collagenase (*MMP9*). (b) H&E staining showed that more monocytes were observed around the residual membrane in a 3-day rat subcutaneous model. * Significant differences compared with the blank group. *, P < 0.05, **, P < 0.01, ***, P < 0.001, ****, P < 0.0001, data without * were not significantly different (NS). Error bars represented mean ± SD.

To determine the effects of SF25 on the immune response, macrophages were stimulated with SF25 extracts for 24 h. The expression of inflammation-related factors (*IL-6*, *TNF-α*, *IL-1β*, *NF-κB1*, and *IL-1rn*) was significantly reduced compared with that in the blank group. Osteoclastogenesis-related genes (*MCSF*) were significantly downregulated compared to those in the blank group. The fibrosis-enhancing factor, *TGF-β1*, was significantly downregulated, whereas *TGF-β3* was significantly upregulated in the SF25 group. The collagenase (*MMP9*) was significantly downregulated in the SF25 group (Figure 7(a)). This decrease in immune response was also noticed using the rat subcutaneous model. H&E staining showed that more monocytes were observed in the F25 group compared with the SF25 group (Figure 7(b)).

### 3.7. Osteogenesis, degradation, and integration evaluation of SF25

**Figure 8.**
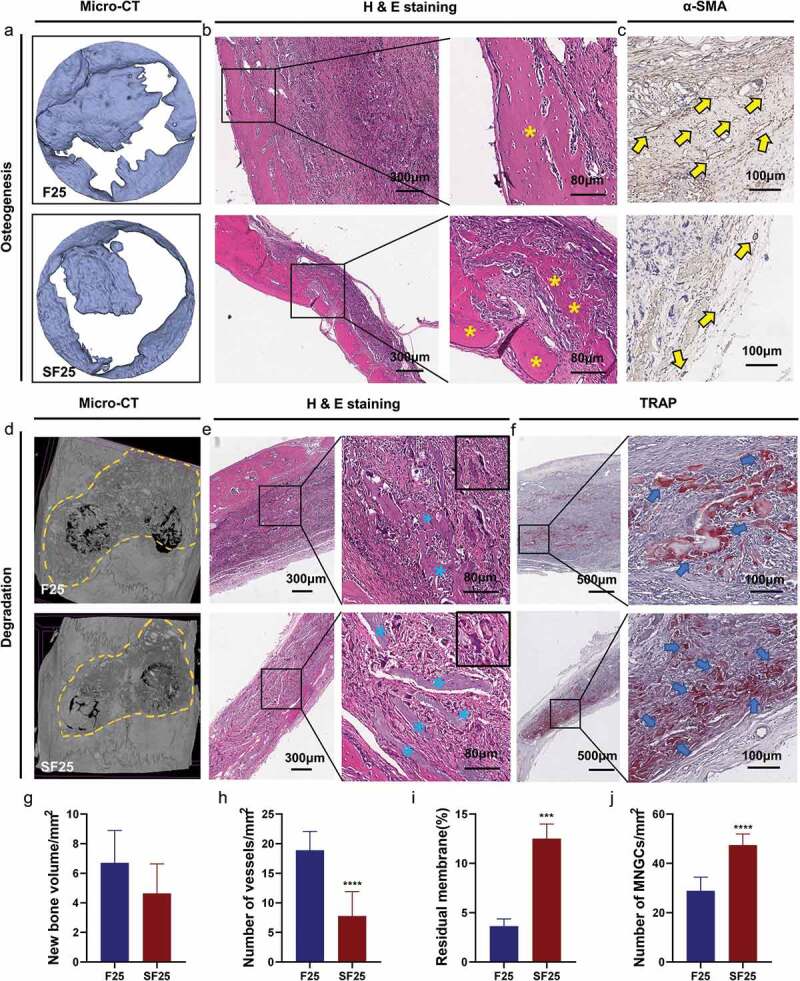
Effects of SF25 on osteogenesis, degradation, and integration properties at the calvarial defect at 4 weeks following surgery. (a) Micro-CT, (b) H&E staining, and (c) α-SMA staining showed newly formed bone formation (yellow asterisks) and blood vessels (yellow arrows) in SF25 compared with F25. (d) Micro-CT, (e) H&E staining, and (f) TRAP staining revealed the residual membrane (blue asterisks), the presence of TRAP+ MNGCs (blue arrows), and the integration property of SF25 compared with those of the F25. Semi-quantitative analysis of (g) new bone volume, (h) the number of vessels, (i) the percentage of residual membrane area, and (j) the number of MNGCs. * Significant differences compared with the blank or F25 groups. *, P < 0.05, **, P < 0.01, ***, P < 0.001, ****, P < 0.0001, data without * were not significantly different (NS). Error bars represented mean ± SD.

In vivo experiments were performed to explore the osteogenesis, degradation, and integration properties of SF25. Newly formed bone lamellae were observed in H&E staining of the SF25 group, whereas the quantitative analyses of micro-CT showed a comparable amount of new bone formation in the SF25 and F25 groups (Figure 8(g)). Regarding the degradation and integration properties, the amount of residual membrane was significantly higher in the SF25 group than that in the F25 group (Figure 8(d,e,i), Supplementary Figure S1). The residual mineralized membrane in the SF25 group presented scattered strips, whereas that in the F25 group presented smaller fragments. More regular fibrous formation with fewer vessels and more TRAP+ MNGCs was found in SF25, whereas vascularized fibrous formation with fewer TRAP+ MNGCs was found in the F25 group (Figure 8(c,f,h,j)).

## 4. Discussion

In the present study, we developed three-phase barrier membranes using fluoride-modified PILP. Three kinds of membranes mediated diverse biological outcomes, which was closely related to ACP-related immunomodulation. The original BCM in the organic phase induced the mildest inflammatory response and degraded mostly after 4 weeks. The organic-inorganic mixture phase of the collagen membrane (F-mBCM) evoked a prominent inflammatory response owing to the F-ACP nanoparticles, resulting in fibrous encapsulation and active angiogenesis, whereas the inorganic phase (SF-mBCM) induced a mild inflammatory response and degraded the least owing to the transition of F-ACP particles into calcium phosphate with low solubility and high crystallinity during sintering.

### 4.1. Fluoride-modified PILP-induced organic-inorganic phase transition of the collagen membrane

The transition from the organic to the inorganic phase via the fluoride-modified PILP method involved two processes. In the BCM stage, the membrane was composed of organic matter. After the PILP process, the membrane was composed of both organic and inorganic matter. After sintering, all organic matter disappeared. These results were confirmed by TG analysis (Figure 6(f)). In contrast, inorganic substances were generated after PILP, with a mixture of both amorphous and crystalline phases (Figures 2(b) and 5(e)). After sintering, increased crystallinity was observed using TEM and XRD (Figure 6(b)). Overall, the composition of the organic substances changed from 100% to 0%, whereas the inorganic matter content increased from 0% to 100%. BCM in an organic phase, F-mBCM with an organic-inorganic mixture phase, and SF-mBCM in an inorganic phase were successfully synthesized through PILP combined with a sintering strategy.

F-ACP nanoparticles were observed for the first time in the present fluoride-modified PILP system (Figure 5(c–e)), mediating subsequent intrafibrillar and extrafibrillar mineralization (Figure 2(a,c)). The formation of F-ACP proved that the process of fluorination simultaneously occurred with mineralization, and the degree of fluorination could affect the mineralization behavior and outcome. As fluoride concentration increased, the degree of extrafibrillar mineralization also increased (Figure 1(a,b)). More crystals were observed around the collagen fibers (Figure 2(c)). This was in accordance with the findings of Saxena et al. [15]. This mechanism may be attributed to the lower solubility of FHA compared with that of HA. Inadequate polymers in the mineral solution were not able to stabilize amorphous precursors from entering collagen fibrils, eventually leading to a higher degree of extrafibrillar mineralization.

### 4.2. Phase transition induced diverse degradation behavior via ACP control

To determine the degradation behavior of F-mBCM and SF-mBCM, *in vivo* experiments were conducted. The results showed that the residuals of F-mBCM and SF25 were higher than those of BCM 4 weeks after surgery, which indicated that the PILP strategy could enhance barrier function (Figures 4(a) and 8(i)). Further analysis showed that, although both F-mBCM and SF25 improved the anti-degradation performance, the integrity of the SF25 membrane was significantly higher than that of F-mBCM (Figure 8(i)). The underlying mechanisms may be closely related to the F-ACP nanoparticles (Figure 5).

ACP can induce aseptic inflammation by mediating macrophage polarization [23]. It could change the morphology of macrophages as well as polarize macrophages into the M1 type, which weakens the osteogenic ability of BMSC. In response to different stimuli, primary macrophages can polarize to a pro-inflammatory M1 phenotype or anti-inflammatory wound-healing M2 phenotype [24]. In the early stages of the inflammatory phase, the inflammatory M1 phenotype is responsible for phagocytic fragmentation, whereas the late M1-to-M2-type transition promotes BMSC osteogenesis and biomineralization [25]. In the F-mBCM group, the expressions of *TNF-α*, *IL-1β*, *IL-6*, and *NF-κB1* were significantly upregulated, revealing a prominent inflammatory response induced by released F-ACP (Figure 5(a)) [26,27]. This proves that macrophages were polarized to the M1 phenotype in the early stage, and further resulted in vascularized fibrous encapsulation and degradation (Figures 3 and 4). On the other hand, after the transition of F-ACP particles into calcium phosphate with low solubility and high crystallinity during sintering, the expression of M1-type related cytokines (*TNF-α*, *IL-1β*, *IL-6*, and *NF-κB1*) was significantly down-regulated in the SF25 group (Figure 7(a)) along with the number of monocytes around the membrane (Figure 7(b)). As a result, a low level of initial immune response avoided the induction of severe vascularized fibrous formation thus optimizing the biocompatibility of the membrane, leading to enhanced barrier performance (Figure 8(d,e,i)). These results collectively indicate that the control of F-ACP plays a central role in mediating diverse inflammatory responses as well as the degradation behavior of the PILP-based membrane.

It should be pointed out that the main observation time points of the *in vivo* model used in this study are 3 days and 4 weeks. However, the biogenic collagen membrane may change the cell composition at other time points and influence the cell state of bone regeneration, such as 6/8 weeks, etc [21,28]. Therefore, further sample evaluation at more time points is still needed to reflect the degradation performance of materials.

### 4.3. Osteogenesis mechanism of fluoride-modified biomineralized collagen membrane

Comparable new bone formation was observed in both F25 and SF25 groups with the BCM group. It should be noted that massive vascularized fibrous encapsulation was accompanied by new bone tissue in the F-mBCM group (Figure 4(b,c)), which may be related to the upregulation of *TGF-β1*, *MMP9*, and pro-inflammatory cytokines (Figure 5(a)). The expression of *TGF-β1* can lead to the formation of fibrous and scar tissue through the activation of the classic TGF-β-Smad-MMP signaling pathway [29,30]. *MMP9* can degrade the extracellular matrix by digesting solubilized collagen I and III monomers [31]. Upregulation of pro-inflammatory cytokines can also induce pre-osteoblasts to differentiate towards a fibroblast phenotype [32] (Figure 5(a)), leading to fibrous encapsulation. In the SF-mBCM group, the expression of *TGF-β1*, *MMP9*, and pro-inflammatory cytokines (*TNF-α*, *IL-1β*, and *IL-6*) were all significantly downregulated (Figure 7(a)), along with reduced angiogenesis and fibrous encapsulation in vivo (Figure 8(c,e)). Additionally, *MCSF* expression was downregulated. *MCSF* can induce osteoclastogenesis by binding to the colony-stimulating factor-1 receptor on macrophages and promoting osteoclast differentiation [33]. Therefore, the downregulation of *MCSF* also promoted bone formation in the SF-mBCM group (Figure 7(a)).

Although fluoride ions promote the osteogenesis performance compared with the blank group to some extent, there is no significant difference between the F-mBCM group and the conventional BCM group, which may be due to the lack of scaffolds with osteoconduction in the bone defect area, such as bone substitute, and the insufficient observation time. Some studies have achieved good osteogenic effects by adding bone substitutes along with barrier membranes [34], and some studies plugged the membrane into the defect as an osteoconduction scaffold itself [35]. Therefore, the use of membrane alone without placing any bone substitute materials to promote osteogenesis still needs further research in the current study model.

### 4.4. Implications of the novel apatite-based barrier membranes

Generally, three strategies were used to prolong the degradation of a collagen membrane. One is to increase the crosslinking or mineralization degree [21], the other is to modulate the phagocytic function of macrophages and foreign body giant cells [36], and the third is to reduce the immunorecognition and related foreign body reaction of the membrane [37]. The current biomimetic mineralization strategy aims to increase the mineralization degree of the membrane. The increase in the collagen membrane’s overall mineralization significantly prolonged the membrane’s degradation process (Figure 4). However, obvious vascularized fibrous encapsulation was noticed, suggesting that excess inflammation existed. Such inflammation is closely related to the inflammatory effect of the release of ACP from biomineralized membrane [38], which is confirmed by TEM (Figure 5). Therefore, the simple biomineralization modification strategy is to some extent contrary to the concept of immunomodulation optimization. Effective control of ACP is key to building novel apatite-based barrier membranes. Future research will focus on the use of preparing organic-coated biomineralized membrane to control the release of ACP and optimize the immune microenvironment. Autologous blood or platelet-rich fibrin (PRF) is an ideal natural wrapping substance with superior biocompatibility. PRF exhibits anti-inflammatory activity and shifts macrophage polarization from the M1 to M2 phenotype [39]. In addition, PRF can decrease the inflammatory response in mesenchymal cells [40]. Cross-linking agents have also shown great potential for regulating target molecule release [41,42], indicating a possible solution for ACP control. These methods may create a favorable environment for tissue regeneration by controlling ACP release.

High-temperature sintering is also an effective way to keep ACP from being released via an amorphous-to-crystalline phase transition. Heat treatment is a common method for stabilizing ACP and improving the crystallinity of HA [43], which affects biological reactions, such as the adsorption of proteins and osteoblast precursor cells [44,45]. We further prepared a completely inorganic phase barrier membrane via the sintering method. By converting ACP into stable crystals, we downregulated the level of membrane inflammation, optimized the immune microenvironment, reduced the degree of vascularized fibrous encapsulation, and further improved the barrier performance. One of the main limitations of heat treatment is that it causes the collagen membrane to solely retain the rigid inorganic scaffold, thereby losing plasticity. Therefore, the sintering strategy has transformation value in non-degradable barrier membranes and personalized barrier stent substitutes.

## 5. Conclusions

F-mBCM and SF-mBCM were prepared to examine the modified degradation behavior of the apatite-based barrier membrane. F-mBCM provoked a strong inflammatory response leading to vascularized fibrous encapsulation owing to the presence of F-ACP nanoparticles, whereas SF-mBCM induced a mild inflammatory response and significantly enhanced barrier performance via eliminating ACP particles. Effective control of ACP is key to building novel apatite-based barrier membranes. A new generation of collagen membranes should be further optimized via ACP control while promoting osteogenic properties.

## Supplementary Material

Supplemental Material

## Data Availability

The data that support the findings of this study are available from the corresponding authors, SW, ZC, upon reasonable request.
