# Supplementary material for: Optimizing the biodegradability and osteogenesis of biogenic collagen membrane via fluoride-modified polymer-induced liquid precursor process
Source: Sci Technol Adv Mater. 2023 Mar 13;24(1):2186690. doi: 10.1080/14686996.2023.2186690 (PMC10013244; doi:10.1080/14686996.2023.2186690)
Supplement: Supplemental Material [file TSTA_A_2186690_SM0110.docx]

Supplementary Material

**Optimizing the biodegradability and osteogenesis of biogenic collagen membrane via fluoride-modified polymer-induced liquid precursor process**

**Xiyan Li^1^, Chuangji Li^1^, Mengxi Su^1^, Xinyi Zhong^1^, Yihan Xing^1^, Zhengjie Shan^1^, Shoucheng Chen^1^, Xingchen Liu^1^, Xiayi Wu^1^, Quan Liu^1^, Ye Li^1^, Shiyu Wu^1*^, Zhuofan Chen^1*^**

*** Correspondence:**

Zhuofan Chen* chzhuof@mail.sysu.edu.cn

Shiyu Wu* [wushy55@mail.sysu.edu.cn](mailto:wushy55@mail.sysu.edu.cn)

**Supplementary Table 1.** Primer pairs used in the RT-qPCR.

| **Gene** | **Primer** |
| --- | --- |
| *GADPH* | Forward: 5’-TCAGCAATGCCTCCTGCAC-3’  Reverse: 5’-TCTGGGTGGCAGTGATGGC-3’ |
| *IL-6* | Forward: 5’-ATAGTCCTTCCTACCCCAATTTCC-3’  Reverse: 5’-GATGAATTGGATGGTCTTGGTCC-3’ |
| *IL-1β* | Forward: 5’-TGGAGAGTGTGGATCCCAAG-3’  Reverse: 5’-GGTGCTGATGTACCAGTTGG-3’ |
| *TNF-α* | Forward: 5’-CTGAACTTCGGGGTGATCGG-3’  Reverse: 5’-GGCTTGTCACTCGAATTTTGAGA-3’ |
| *NFκB1* | Forward: 5’-GCGCTGTGGCTGAAATAATCG-3’  Reverse: 5’-GCTACACTCAGATCGCTCCA-3’ |
| *IL-1rn* | Forward: 5’-ACCAAATATCAAACTAGAAGAAAA-3’  Reverse: 5’-CAGAGCGGATGAAGGTAA-3’ |
| *TGF-β1* | Forward: 5’-GTGGAAATCAACGGGATCAGC-3’  Reverse: 5’-CAGCAGTTCTTCTCTGTGGAGC-3’ |
| *TGF-β3* | Forward: 5’-GCGCTGTGGCTGAAATAATCG -3’  Reverse: 5’-GCTACACTCAGATCGCTCCA -3’ |
| *MCSF* | Forward: 5’-AGCAGAACAAGGCCT GTGTC-3’  Reverse: 5’-AAGCTGTTGTTGCAGTTCTTGG-3’ |
| *VEGF* | Forward: 5’-GTCCCATGAAGTGATCAAGTTC-3’  Reverse: 5’-TCTGCATGGTGATGTTGCTCTCTG-3’ |
| *MMP9* | Forward: 5’-ACCTCCAACCTCACGGACA-3’  Reverse: 5’-AGGTTTGGAATCGACCCACG-3’ |

**
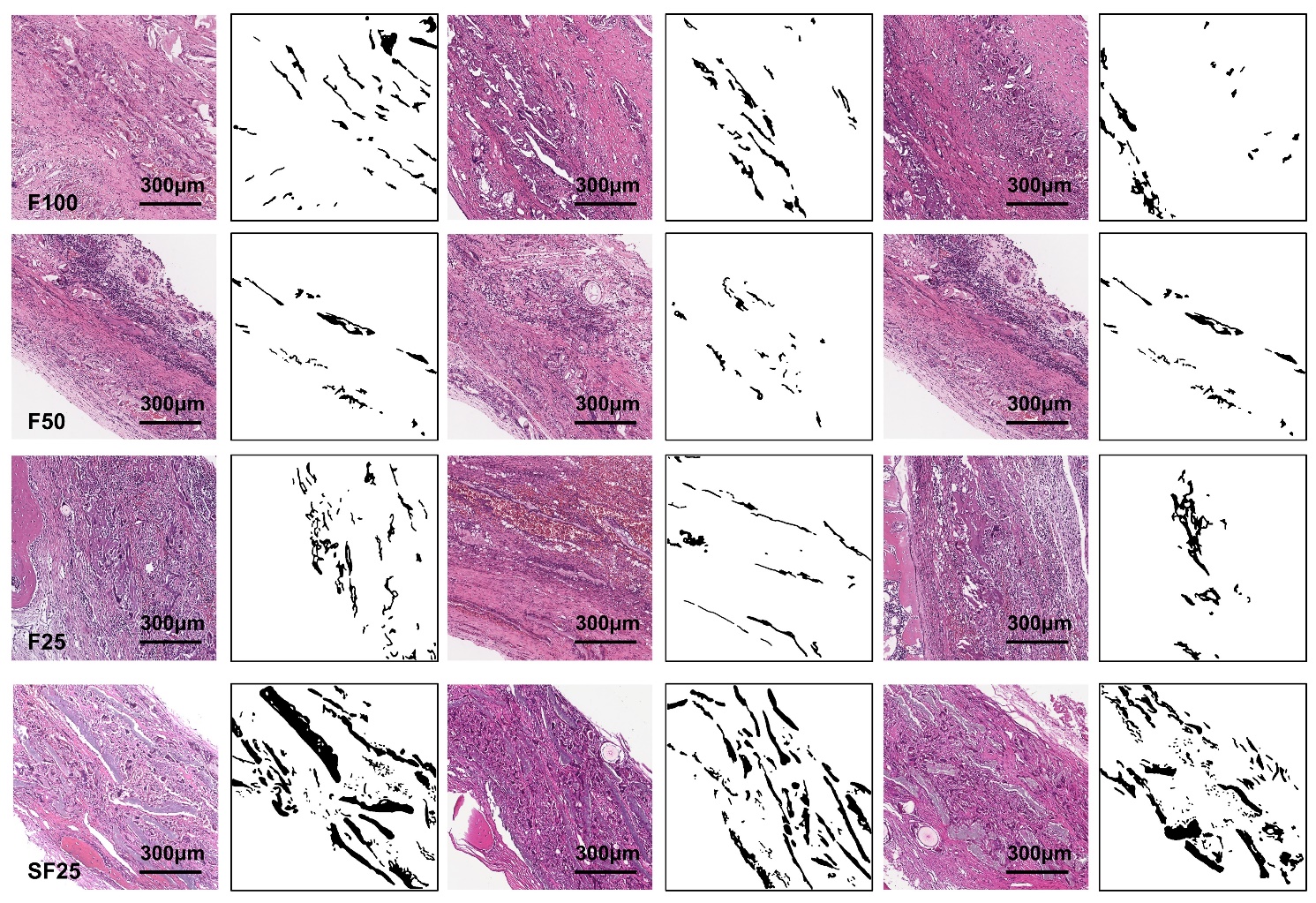
**

**Supplementary figure 1.** The residual membrane of F-mBCM and SF25 after 28 days implantation in a rat calvarial defect model according to H&E staining.
